# Safety and adherence of early oral immunotherapy for peanut allergy in a primary care setting: a retrospective cross-sectional study

**DOI:** 10.1186/s13223-024-00916-5

**Published:** 2024-10-24

**Authors:** Victoria Landry, Rachel Lewis, William Lewis, Lyndsey MacDonald, Beth Carson, Kavish Chandra, Jacqueline Fraser, Andrew J. Flewelling, Paul Atkinson, Chris Vaillancourt

**Affiliations:** 1 Dalhousie University, Halifax, NS Canada; 2 NB Allergy Associates, Saint John, NB Canada; 3 Saint John Regional Hospital Emergency Medicine, Saint John, NB Canada; 4 Horizon Health Network Research Services, Saint John, NB Canada; 5 106 Neil St., Rothesay, NB E2H 1J6 Canada

**Keywords:** Oral immunotherapy, Primary care, Preschool, Peanut allergy

## Abstract

**Background:**

Peanut allergy is a common food allergy with potentially life-threatening implications. Early oral immunotherapy for peanut allergy (P-EOIT) has been shown to be effective and safe in research and specialty clinic settings. Provision of P-EOIT in primary care would make it available to more patients. We sought to assess the safety of P-EOIT in a primary care setting by documenting the rates of peanut-related allergic reactions leading to emergency department (ED) visits and use of epinephrine. We also examined adherence by assessing the percentage of patients reaching maintenance phase and continuing ingestion after one year of P-EOIT.

**Methods:**

This retrospective study included all patients aged less than 36 months who started P-EOIT at a primary care allergy clinic in New Brunswick, Canada, from 2016 to 2020. The population included patients who (1) had a history of an allergic reaction to peanuts with a positive skin prick test or positive peanut specific IgE level (ps-IgE) or (2) no history of ingestion and a baseline ps-IgE ≥5 kU/L. Patients had biweekly clinic visits with graded increases in peanut protein up to a maintenance dose of 300 mg of peanut protein daily. A blinded retrospective review of paper charts and electronic medical records was conducted along with phone interviews regarding ED visits and epinephrine use.

**Results:**

All 69 consented patients reached maintenance dose over a median of 29 weeks, and 66 patients (95.7%) were still regularly consuming peanut protein after 1 year of maintenance. One patient had a peanut ingestion-related ED visit requiring epinephrine during the escalation phase of peanut protein dosing (1.4%). During the first year of maintenance phase, no patients had peanut ingestion-related ED visits nor required epinephrine.

**Conclusion:**

Early oral immunotherapy for peanut allergy in a primary care setting appears to be safe and our findings suggest that it does not lead to an increased burden of emergency department visits. Our population had high adherence rates, with the majority achieving maintenance dose and staying on this dose for one year.

## Background

Food allergy prevalence is on the rise and currently affects an estimated 8% of children, with peanut allergy accounting for 25.2% of childhood food allergies [1–3]. Peanut allergy is a relatively common IgE-mediated food allergy with potentially life-threatening implications, affecting an estimated 2% of children, [1, 4, 5]. Until recently, the standard of care for peanut allergy in most of North America was avoidance of peanuts with reactive treatment for exposures [6, 7]. Contrary to former recommendations for delayed exposure, early introduction of peanut products at 4–11 months of age is the current guideline for prevention of peanut allergy, and screening prior to exposure is not needed [1, 2, 8–11]. Oral immunotherapy (OIT) is an exciting new treatment for food allergy [7, 8, 12] and consists of daily ingestion of the offending allergen, with dose increases over time to improve clinical tolerance to the food [13]. The goal of OIT is desensitisation, defined as an “increased allergic reaction threshold while on therapy” [14, 15]. Once the target dose is reached, the patient is protected from developing anaphylaxis during accidental exposure as long as the allergen is regularly consumed [16]. Current research is investigating the possibility of sustained unresponsiveness, defined as “absence of clinical reactivity after discontinuing therapy for short periods of time, typically 4–8 weeks”, and remission, defined as “a state of non-responsiveness after discontinuation of immunotherapy” [14].

Early oral immunotherapy for peanut allergy (P-EOIT) has been shown to be effective and safe in specialist and research settings [5, 17]. The safety and effectiveness of P-EOIT was demonstrated in the landmark study by Vickery et al. in 2017. In a population of children aged 9–36 months, 81% of patients were desensitized at the end of treatment, and 78% of patients achieved sustained unresponsiveness after stopping therapy for 4 weeks, with only mild to moderate reactions in the majority [5]. Several trials have indicated that starting immunotherapy in infants and preschoolers while the immune system is maturing and pliable might be more effective and safer than starting later due to the immunomodulatory effect of oral immunotherapy [4, 13, 14, 17]. Remission has been shown to be associated with younger age at starting OIT [18]. A post-hoc analysis of the IMPACT trial suggested an inverse relationship between age and remission outcome, with 71% of remission cases noted in those <24 months old receiving peanut oral immunotherapy, suggesting that the enhanced window for remission closes very early [14]. This is in contrast to a 19% remission rate in those aged 36–47.9 months, which is similar to the 20% rate of sustained unresponsiveness found in the POISED study [19]. A 2022 paper demonstrated that peanut OIT has equal effectiveness and may be safer in infants less than 12 months old compared with non-infant preschoolers, with infants having no severe reactions, no epinephrine use, and fewer reactions [13]. In addition to these efficacy and safety findings, early introduction of peanut OIT appears to be more cost-effective than waiting to initiate therapy in older children. A recent study showed that real-world use of P-EOIT in Canada had lower cost and higher quality of life than avoidance therapy [18, 20].

Many Canadian patients are unable to access healthcare support for their food allergy, and currently there is a lack of capacity to treat all eligible patients with peanut early oral immunotherapy in specialist settings [13, 16]. In Canada, there are under 250 allergists to provide care for an estimated 6% of the population living with food allergies (roughly 3 million people) [3]. Lack of allergists is compounded by a lack of oral immunotherapy provision, with a reported 52% of allergists providing OIT in Canada [21]. Patients in rural settings are disproportionately affected by this lack of access to allergist care, as the majority of specialists are located in urban centres [22]. More young patients would be able to access OIT if it could be safely delivered by primary care clinicians such as paediatricians and family physicians who are already providing allergy care in under-serviced areas [16]. If primary care clinicians focused on OIT introduction at early ages, this would provide early OIT to more infants, while decreasing waitlists for allergists and allowing them to focus on higher-risk infants and older children [23]. Ideally, primary care provision of OIT in underserved areas would be offered in collaboration with an experienced allergist [23]. There is currently a lack of literature assessing whether P-EOIT can be performed safely in a primary care setting.

The current study was conducted in the province of New Brunswick in Canada. New Brunswick has a population of 812,000 and has no practicing residency-trained pediatric allergy specialist providing OIT. The closest speciality allergy clinic providing OIT is in the adjacent province of Nova Scotia. In 2015 a group of family physicians providing allergy care in New Brunswick sought to investigate if they could safely provide early peanut OIT. They reviewed the current literature and attended several OIT and oral food challenge (OFC) training sessions provided by the American College of Allergy, Asthma and Immunology (ACAAI). They also upgraded their anaphylaxis care plan for patients receiving OIT and developed a dedicated OIT and OFC assessment area. This primary care allergy centre offers clinics in various locations of New Brunswick, making it more convenient for patients to attend regular OIT visits.

## Methods

### Aim

The current study seeks to identify if provision of P-EOIT in a primary care setting results in increased rates of peanut-related anaphylaxis requiring epinephrine injection, or increased burden of emergency department (ED) visits for allergic reaction during escalation and maintenance of immunotherapy. This study also examines patient uptake and adherence to P-EOIT.

### Setting

This single-center blinded retrospective study was carried out with approval from the local research ethics board [24, 25]. Verbal informed consent was obtained by phone from patients’ substitute decision maker. Study participants included patients at a primary care allergy clinic in New Brunswick, Canada, with offices in Saint John, Oromocto and Waterville. These clinics provide care to patients throughout the province and began offering early oral immunotherapy for peanut allergy in 2016. This allergy program has excellent support both from the New Brunswick Medical Society and the Provincial Government. In November 2021 this clinic successfully negotiated the first Oral Immunotherapy Billing Codes in Canada. Of note there is a fee structure differential based on speciality or primary care billing codes.

The protocol for OIT administration is outlined in Appendix 1 and was modeled after recent P-EOIT studies [5, 14, 17].

### Patient selection

Participants included all patients starting peanut oral immunotherapy at 6–36 months of age from 2016 to 2020. Patients were enrolled if they had either (1) a history consistent with an allergic reaction to peanut occurring within 60 min of peanut ingestion with either a positive skin prick test (SPT) wheel diameter of 3 mm or more, or a positive peanut specific IgE (ps-IgE) level of 0.35 kU_A_/L or more, or (2) a baseline ps-IgE of 5 kU_A_/L or more (performed based on family history of peanut allergy or other food allergies) with no personal history of peanut ingestion. Consistent with prior studies, oral food challenges were not performed on all patients due to resource constraints and the delay to enrollment that this would cause [5, 17]. Children were excluded if they had a previous life-threatening anaphylactic reaction to peanut with hypoxia, hypotension, or altered level of consciousness, or severe atopic dermatitis requiring systemic therapy, or uncontrolled asthma requiring more than medium-dose inhaled corticosteroid therapy.

### Procedures

Starting in 2016 all new referrals to the allergy clinic regarding possible peanut allergy were triaged as urgent and every effort was made to assess them within two weeks. As part of a patient-centered shared decision model, substitute decision makers of children with newly diagnosed peanut allergy were offered the option of peanut avoidance or P-EOIT. The benefits and risks of both treatment modalities were discussed. Those consenting to P-EOIT were given written and verbal instructions regarding identification and treatment of common side effects and when to withhold a peanut protein dose. They were also given instructions on how to deal with mild and severe allergic reactions including anaphylaxis (see Appendix 2). Of note, this handout was transitioned to advising the use of a non-sedating anti-histamine instead of diphenhydramine as recommendations changed, and the handout from Soller et al. was used instead [26]. Each patient was prescribed an epinephrine auto-injector and provided a demonstration on its use.

The protocol for peanut early oral immunotherapy closely followed the low dose protocol outlined in Vickery et al.’s 2017 study [4]. It consisted of graded increases in peanut protein dose every two weeks, after the initial two escalation days one week apart (Appendix 1). Patients had the option of using Bamba peanut puffs, peanut flour mixed into food, or a hybrid of the two. The target maintenance dose was 300 mg of peanut protein daily. Each escalation in dose of peanut protein was performed in the clinic and the patient was observed for a minimum of 30 min post consumption to monitor for any adverse reactions. Between clinic visits patients ingested the specified dose of peanut daily at home. Families were asked to note symptoms at home and report them to their care provider at the next visit. Symptoms and management of allergic reactions during clinic buildups, including epinephrine use, were recorded in the patient’s chart and later graded according to the World Allergy Organization Subcutaneous Immunotherapy Systemic Reaction Grading System [27]. Any severe adverse reactions, use of epinephrine, and visits to an emergency department between clinic appointments were also documented in patient charts at each visit. Patients were recommended to continue consuming peanut protein daily after maintenance dose was reached. Ps-IgE was repeated at one year after starting immunotherapy, and yearly follow up was continued for all patients. After one year of maintenance parents were advised to slowly increase the home dose to an equivalent of one tablespoon of peanut butter daily over a year, and then two tablespoons daily over the subsequent year. After increasing their daily dose (1–2 years after reaching maintenance), patients who continued to ingest peanut protein daily were advised they could decrease frequency of consumption to 3 times a week as long as they were tolerating ingestions. Clinic charts were extensively reviewed when patients reached 5 years of age. If patients were consuming at least two tablespoons of peanut butter or five grams of peanut protein from another source at least 3 times a week, they were advised to continue this practice as long as they were tolerating the ingestions. It was explained to them that if they stopped ingesting peanut protein for an extended period (at least 2–3 months), or if they were ingesting below three grams of peanut protein at a time, then an oral food challenge would be recommended.

Volunteers blinded to study objectives collected data from patient charts and entered it into a standardized data collection sheet [16]. Patient families were then contacted by phone by our blinded volunteers to inquire about use of epinephrine and emergency department (ED) visits related to peanut allergy during escalation and maintenance phases of immunotherapy. A standardized questionnaire was used during this phone conversation (Appendix 3). Each patient’s provincial electronic medical record was reviewed to identify any peanut allergy-related visits to a New Brunswick emergency department that were not captured in the patient’s paper chart or in the phone interview. For ED visits with insufficient information, ED paper charts were also reviewed to determine whether the visit in question was related to peanut allergy.

### Statistical analysis

To evaluate safety, we examined the proportion of patients requiring epinephrine or visiting an emergency department due to peanut-related allergic symptoms during escalation and maintenance phases of peanut protein ingestion. To evaluate adherence, we looked at the percentage of patients reaching maintenance dose, as well as the percentage of patients still ingesting peanut protein after a year of maintenance therapy. Using Excel spreadsheet functions, percentages were calculated for categorical variables, and medians with interquartile ranges were calculated for continuous variables.

## Results

### Baseline characteristics of patients

**Fig. 1:**
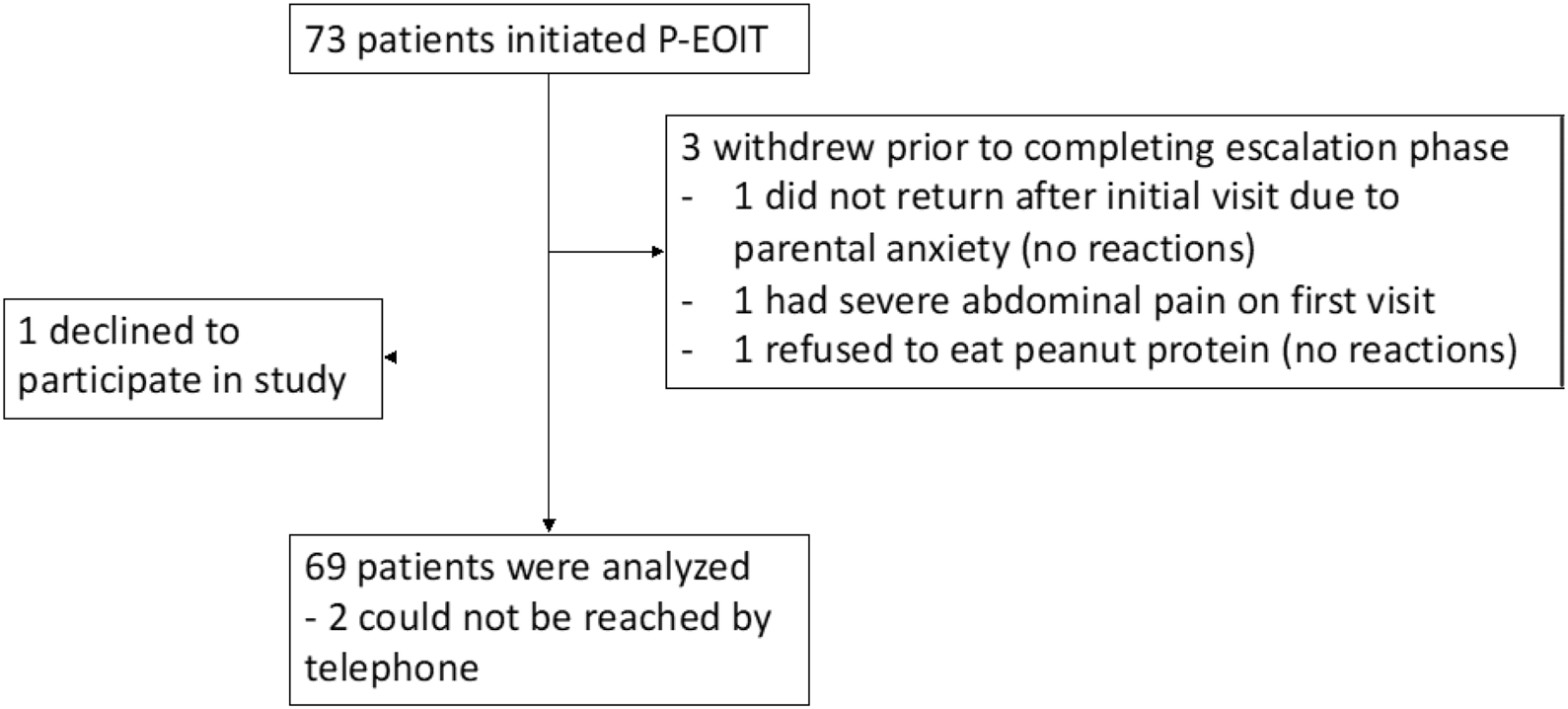
Trial profile

From October 2016 to September 2020, 72 eligible preschoolers were started on peanut early oral immunotherapy (the intention to treat group). Three withdrew from P-EOIT prior to reaching maintenance dose, leaving 69 patients to be analyzed (the per protocol group) (Fig. 1). Median age at P-EOIT start was 13 months (IQR 8) and the majority were white (84.1%) males (55.1%). Most patients (94.2%) had experienced a previous reaction to peanut with the majority being mild (grade 1, 71%) and some moderate (grade 2, 23.2%) reactions (Table 1). No grade 3 or 4 reactions were reported. Median baseline ps-IgE level was 1.78 kU_A_/L (IQR 3.98) and median SPT wheal diameter was 8 mm (IQR 4). Four patients (5.8%) were never exposed to peanut before entry into P-EOIT and their median baseline ps-IgE level was 3.55 kU_A_/L (IQR 19.6). One of these had an egg and tree nut allergy and declined OFC. Two others had positive SPTs (8 mm and 15 mm) performed due to parental anxiety about introducing peanut (one had a sibling with peanut allergy and the other had a milk allergy), but declined OFC. These patients requested OIT specifically. The fourth patient had a ps-IgE of 28 kU_A_/L and was referred for OIT by an allergist in another province. Four other patients with convincing histories of allergic reaction to peanut had negative SPTs, but these patients either had positive ps-IgE or were offered an OFC. Two patients under 1 year of age had negative ps-IgEs but had convincing histories of allergic reaction to peanut and positive SPTs of 7 mm and 6 mm. After 1 year of OIT both patients had a ps-IgE of 0.13 kU_A_/L. Most patients had other atopic conditions (81.1%) with the majority having eczema (78.3%). Many patients (53.6%) had other food allergies with the majority having egg allergy (44.9%). A minority of patients (10.1%) were simultaneously undergoing OIT for another food allergy at the same clinic.

**Table 1:** Baseline characteristics of patients undergoing P-EOIT (*N* = 69 per protocol)

Sex: male, *n* (%)	38 (55.1)
Age (months) at entry into OIT, median (IQR)	13 (8)
≤ 12 months, *n* (%)	32 (46.4)
13–24 months, *n* (%)	34 (49.3)
25–36 months, *n* (%)	3 (4.3)
Other atopic conditions, *n* (%)	56 (81.1)
Eczema	54 (78.3)
Asthma	6 (8.6)
Allergic rhinitis	1 (1.4)
Sibling with peanut allergy, *n* (%)	4 (5.8)
Other food allergies, *n* (%)	37 (53.6)
Egg	31 (44.9)
Milk	10 (14.5)
Sesame	7 (10.1)
Tree nut	5 (7.2)
Initial allergic reaction to peanut, *n* (%)	65 (94.2)
Age (months) at initial reaction, median (IQR)	8 (5)
No. of months between peanut reaction and first OIT dose, median (IQR)	3 (3)
Baseline peanut SPT in mm, median (IQR)	8 (4)
No data, *n* (%)	6 (8.7)
Negative, *n* (%)	4 (5.8)
Baseline peanut specific IgE level, kU_A_/L, median (IQR)	1.78 (3.98)
No data, *n* (%)	1 (1.4)
<0.1 kU_A_/L, *n* (%)	2 (2.9)
Referral source, *n* (%)	
Family physician	42 (60.9)
Pediatrician	11 (15.9)
Emergency department	8 (11.6)
Allergist	4 (5.8)
Nurse practitioner	4 (5.8)
Peanut protein type used in buildup, *n* (%)	
Bamba-only	5 (7.2)
Peanut flour-only	7 (10.1)
Hybrid	57 (82.6)

### Adherence

Of 73 patients who began P-EOIT, three withdrew prior to completing the escalation phase, leaving 69 patients to include in the analyses (Fig. 1).

Of the 69 patients consented and analyzed per-protocol, 100% reached the maintenance dose of 300 mg peanut protein per day, over a median of 29 weeks (IQR 5.5). These 69 patients represent 94.5% of the initial 73 in the intention-to-treat population.

At 1 year after start of P-EOIT, median ps-IgE level was 0.84 kU_A_/L (IQR 3), compared with the baseline median ps-IgE of 1.78 kU_A_/L (Table 2). Twelve (17.4%) patients had negative ps-IgE at 1 year of follow up. The majority of patients (66, 95.7%) were still ingesting peanut protein daily after 1 year of maintenance, with most using peanut butter (44.9%) or Bamba (40.6%) as their source of peanut protein.

**Table 2:** Adherence data (*N* = 69 per protocol)

Patients who reached maintenance dose	69 (94.5% of 73 intention to treat; 100% of 69 per protocol)
Weeks to reach maintenance dose (median, IQR)	29 (5.5)
ps-IgE at 1 year after starting OIT (median, IQR)	0.84 kU_A_/L (3)
Type of peanut protein used for maintenance (*n*, %)	
Bamba	28 (40.6)
Peanut butter	31 (44.9)
Peanut flour	6 (8.7)
Peanuts	3 (4.3)
Bamba + peanut butter	1 (1.4)
Still ingesting peanut protein after 1 year of maintenance (*n*, %)	66 (95.7)

### Safety

#### Phone interviews

The majority of patients (67, 97.1%) successfully completed phone interviews for the study. According to family recall during phone interviews, one patient (1.4%) had an ED visit and required epinephrine for a reaction to peanut. Two families reported sesame allergy-related epinephrine use for their children, neither of which involved exposure to peanut. One of these children had difficulty breathing and urticaria, and the other had difficulty breathing, swelling, erythema and itch.

#### Provincial EMR ED visits (Table 3)

**Table 3:** ED visits as per EMR and chart review

	Total visits (*n*, %)	During escalation (*n*, %)	During maintenance (*n*, %)
Total ED visits	92	53 (57.6)	39 (42.4)
Admissions (included in ED visits)	2 (2.2)	1 (viral induced urticaria)	1 (asthma)
Infection	62 (67.4)	38 (71.7)	24 (61.5)
Pneumonia	3 (3.3)	1 (1.9)	2 (5.1)
Fever	9 (9.8)	6 (11.3)	3 (7.7)
URTI^a^	25 (27.2)	17 (32.1)	8 (20.5)
Urinary^a^	4 (4.3)	2 (3.8)	2 (5.1)
GI^a^	6 (6.5)	3 (5.6)	3 (7.7)
Skin^a^	9 (9.8)	6 (11.3)	3 (7.7)
Otitis Media^a^	6 (6.5)	2 (3.8)	4 (10.3)
Minor injury^a^	13 (14.1)	6 (11.3)	7 (17.9)
Wheeze^a^	8 (8.7)	4 (7.5)	4 (10.3)
Miscellaneous^a^	5 (5.4)	3 (5.6)	2 (5.1)
Allergy^a^	4 (4.3)	3 (5.6)	1 (2.6)

Wheeze and urticaria cases had no temporal relationship to peanut OIT

URTI: pharyngitis, tonsillitis, croup, sinusitis, URTI, unspecified respiratory infection; Urinary: UTI, swollen penis, oliguria; GI: gastroenteritis, norovirus; Skin: cellulitis, impetigo, red swollen ear, HFMD, viral exanthem, and viral induced urticarial; Otitis media: AOM, earache; Minor injury: minor head injury, superficial head injury, radial head subluxation, lower extremity injury, elbow injury, concussion, buckle fracture, cast check, head injury; Wheeze: asthma, wheeze, bronchiolitis; Miscellaneous: constipation, conjunctivitis, teething, lymphadenopathy, blood in stool; Allergy: hives and allergic reaction

^a^ED visit reasons

According to provincial EMR data and review of ED paper charts, forty-two patients (60.9%) had a total of 92 ED visits, most of which (57.6%) occurred during escalation phase of P-EOIT. Most were infection-related visits (67.4%), with upper respiratory tract infection (URTI) being the most common visit reason (27.2%).

Four patients (5.8%) each had one allergy-related ED visit identified through the provincial EMR. One of these (1.4%) was related to peanut ingestion. It occurred during a febrile viral illness during escalation phase of P-EOIT. The patient vomited 15 min after ingestion of their home dose of 95 mg of peanut protein, and the parents also felt there was wheezing involved. The patient received one dose of epinephrine in the ED.

The three remaining ED visits were unrelated to peanut ingestion. One was urticaria after accidental exposure to milk in a patient with known milk allergy, one was urticaria related to viral illness vs. amoxicillin exposure, and one was a reaction to hazelnut which required epinephrine. This reaction occurred after the patient’s first exposure to hazelnut, several hours after peanut protein ingestion during escalation phase, and consisted of diffuse hives and angioedema of the lips.

In summary, through review of clinic charts, telephone interviews and the provincial electronic medical record, two patients were ultimately found to have used epinephrine, both of whom went to the ED. One of these was related to peanut ingestion and occurred during the escalation phase of peanut protein OIT (1.4%). During the first year of maintenance phase, no patients had peanut ingestion-related ED visits nor required epinephrine.

## Discussion

To our knowledge this is the first study examining early oral immunotherapy for peanut allergy in a non-specialist setting. We were able to recruit very young children into the program with a median age of 13 months starting OIT, and baseline median ps-IgE of 1.78 kU_A_/L. This was a lower age and median ps-IgE than in the IMPACT trial (39 months, ps-IgE 53.1 kU_A_/L) [14], the Real-World trial (23 months, ps-IgE 5.03 kU_A_/L) [17], and the Vickery trial (28.5 months, ps-IgE 14.4 kU_A_/L) [5]. Our low median age at initiation of OIT is one of the strengths of this study, with 46% of patients starting before one year of age. By prioritizing infant allergy referrals, we were able to assess new peanut allergy consults in a timely fashion, keeping wait times short and thus seeing patients at young ages with low initial ps-IgE levels and SPTs when they are just starting to acquire peanut allergy.

Our high adherence rate reflects the high tolerability of this treatment at very young ages. In our cohort, 69 of 73 patients (94.5%) completed escalation and reached maintenance phase. Of those who completed escalation, 66 (95.7%) continued to regularly ingest peanut protein after 1 year of maintenance. Our adherence rates are similar or slightly higher than in previous literature. In the IMPACT trial, 73% completed escalation and maintenance [14]. In the Real-World trial 90% of patients reached maintenance phase, and 1.83% of these patients dropped out during maintenance phase [17]. In the Vickery study, 86.5% of patients completed the OIT protocol [5].

Our measurements of safety were similar to previous studies [5, 14, 17]. One patient in our study had a peanut-related grade 2 (moderate) reaction requiring epinephrine and an emergency department visit during escalation phase. This equates to 1.4% of our per-protocol patients, similar to studies in real-world specialty clinics. We did not see any patients with grade 3 or 4 reactions. There were no diagnoses of eosinophilic esophagitis among our patients during escalation or maintenance phases. The majority of our peanut-allergic patients had eczema (78.3%) which is consistent with research demonstrating a high rate of peanut allergy in those with atopic dermatitis [10, 28]. Similar to previous studies, most of our patients experienced only minor side effects. Though we did not explicitly examine incidence of minor side effects, we have no evidence to suggest that any of our patients experienced a reaction where epinephrine or an ED visit was warranted but such care was not provided.

In comparison, the IMPACT trial showed epinephrine use in 22% of participants, most of which occurred during maintenance phase, with the majority being grade 2 reactions [14]. In Vickery’s study, one patient (2.7%) required epinephrine during escalation phase [5]. In the Real-World trial, 4.1% of patients received epinephrine (11 of 12 reactions were grade 2), and 1.1% (3 patients) had an ED visit for allergic reaction (all were grade 2) during escalation phase [17]. During maintenance phase, 10 patients (8.1%) had grade 1 allergic reactions, three (2.4%) had grade 2 reactions, and two patients (1.6%) received epinephrine associated with peanut ingestion, with one (0.8%) patient transferred to the ED [29]. Our rates of epinephrine use were slightly lower than in the Real-World study [17], which may due to our lower median age of enrollment. In contrast to these relatively low rates of epinephrine use, a 2014 OIT study among patients 3–24 years old found that 6% of patients on maintenance required epinephrine [30], and a 2018 study reported 63 epinephrine-treated reactions in 28 patients (40%) during the first 6 months on maintenance (and 57% in the first year) [31]. These higher rates are postulated to be due to older ages at OIT initiation. Future studies are needed to establish whether lower rates of epinephrine use as observed in our study are related to younger ages at OIT initiation.

The Vickery trial and Real-World Safety trial showed that starting OIT for peanut allergy in young children 9–71 months old was safe and had potential for enhanced effectiveness compared with starting at older ages [5, 17]. The IMPACT trial showed that P-EOIT induces remission in the youngest children and may be safer in infants than in non-infant preschoolers [14]. There is also evidence that younger patients have higher rates of remission than their older counterparts though it currently remains unknown whether this remission is lifelong [29].

In a recent survey, only 52.2% of allergists provided OIT, citing among other barriers a lack of safety and efficacy data [6]. Based on the IMPACT trial, we do know that as the patient’s age increases so does the incidence of more allergic reactions during OIT [14]. Our study adds to the growing evidence that initiating peanut OIT at younger preschool ages is safe and potentially better tolerated than at older ages [13, 17]. These findings reinforce the importance of having timely access to P-EOIT for infants with peanut allergy.

### Study limitations

Our low rates of systemic reactions could be attributed to the less severe phenotypes seen in this clinic, which is most likely due to the low median starting age at OIT facilitated by the low wait times after referral for suspected allergy. It is possible that in these cases re-incorporating small doses of peanut protein into the child’s regular diet could have led to similar outcomes, but formalizing the process through OIT is likely safer and associated with higher adherence rates than informal advice to re-incorporate peanut into the diet (further studies would be needed to confirm this theory). Many of these families were very anxious about re-incorporating peanut into their child’s diet, again pointing to the value of guidance and supervision through OIT.

Not all patients enrolled in our primary care P-EOIT program underwent an entrance OFC due to resource limitations and a high risk of reaction. It is therefore possible that some patients did not in fact have a clinical peanut allergy, despite having a history consistent with an allergic reaction to peanut and objective sensitization. This may potentially skew our results toward an increased appearance of safety. However, the practice of not requiring an entrance OFC is consistent with recent real world studies of pre-schoolers undergoing OIT for peanut, tree nut or sesame allergy [17, 32]. In addition, in studies where OFCs were performed, the majority of patients failed the challenge with a significant number requiring epinephrine, and the OFCs rarely changed the decision to undergo OIT [5, 14].

Patients with risk factors but without a history of allergic reaction to peanut were either offered OFC or initiated P-EOIT without OFC based on a high ps-IgE. The decision to initiate OIT was made together with substitute decision makers after discussing the risks and benefits of peanut avoidance vs. OIT. These situations can be challenging for clinicians to navigate, because though we have good predictive parameters to assess which patients will have a persistent peanut allergy at 4 years of age, we have less useful parameters to predict which patients will become tolerant [33].

The retrospective nature of our study may have led to difficulty with memory on the substitute decision makers’ part during phone interviews, especially since some parents were asked to recall as far back as three years. This recall bias was mitigated by the blinded review of clinic charts and electronic medical records for emergency department visits. Parents’ recollection was overall congruent with the clinic chart notes and EMR data. It is possible that a patient could have attended an ED outside the province of New Brunswick without our knowledge, as we only reviewed the provincial EMR. However, there was no documentation by clinic charts or phone interviews to support this.

We do not currently have evidence-based guidelines on what the training requirements should be for non-residency-trained allergy specialists to deliver P-EOIT safely. Interestingly, in a recent Canadian Specialty Allergist survey examining barriers to OIT, 55% of allergy specialists performing OIT reported no formal training in it [21]. The primary care physicians in our group critically reviewed current literature and attended OIT and OFC workshops in preparation to deliver P-EOIT. If we are to meet the needs of patients and effectively respond to the call to offer salvage OIT as soon as possible after failed primary prevention of peanut allergy [7], decisions must made as to how this can be accomplished. A recent article by world leaders in OIT suggested that OIT-trained allergists should form partnerships with and train local physicians in underserved areas [34]. Now may be the time for collaboration of formally trained allergists with other health care providers interested in allergy medicine (family physicians, pediatricians and perhaps nurse practitioners) to increase access to OIT for low-risk children in rural and underserved areas with inadequate access to FRCPC-trained allergists.

## Conclusions

This blinded retrospective study demonstrates that provision of peanut early oral immunotherapy in a primary care setting is safe with high adherence rates. Only one patient had a peanut reaction-related ED visit with epinephrine use during escalation phase, and no patients had severe peanut reactions during maintenance phase. Completion rates of oral immunotherapy were excellent as were continuation rates of maintenance dosing for at least 1 year. Thus, early oral immunotherapy for peanut allergy in a primary care setting appears to be safe with high adherence rates and does not seem to lead to an increased burden of emergency department visits.

## Data Availability

Data is provided within the manuscript or supplementary information files. The datasets used and/or analysed during the current study are available from the corresponding author on reasonable request.

## Appendix 1: P-EOIT protocol

This table shows peanut protein dose increases over time, with the initial dose being 5–7 mg. All dose escalations were performed in clinic with a minimum observation period of 30 min post ingestion. The patient continued to consume the specified daily peanut protein dose between clinic visits. The dosages varied slightly based on the type of peanut protein used (flour or Bamba).

**Table:**

Week no.	Dose (mg)	Interval (weeks)	% Increase
1	5–7		
2	10–13	1	100
4	20–27	2	100
6	40–53	2	100
8	60–66	2	50
10	80–83	2	33
12	100–99	2	25
14	120–133	2	20
16	166–160	2	38
18	199–200	2	20
20	232–240	2	17
22	265–320	2	28
24	298	2	12.5
26	331	2	11

## Appendix 2: Patient instructions

.

## Appendix 3: Phone questionnaire

.

## Abbreviations

OIT: Oral immunotherapy
P-OIT: Peanut oral immunotherapy
P-EOIT: Peanut early oral immunotherapy
ED: Emergency department
URTI: Upper respiratory tract infection
EMR: Electronic medical record
UTI: Urinary tract infection
GI: Gastrointestinal
HFMD: Hand foot mouth disease
AOM: Acute otitis media
ps-IgE: Peanut-specific immunoglobulin E
IQR: Interquartile range
SPT: Skin prick test
ACAAI: American College of Allergy, Asthma and Immunology
